# Identification of key COVID-19 infection contexts over time and the importance of within household infections

**DOI:** 10.3205/dgkh000678

**Published:** 2026-09-15

**Authors:** Julia Hurraß, Sydney Paltra, Anna Wolff, Barbara Grüne, Sebastian Müller, Annelene Kossow, Johannes Nießen, Kai Nagel

**Affiliations:** 1 HYGIUM – Center for Hygiene and Environmental Medicine, Cologne, Germany; 2 Institute for Translational Research, CECAD Cluster of Excellence, University of Cologne, Cologne, Germany; 3 Technische Universität Berlin, FG Verkehrssystemplanung und Verkehrstelematik, Berlin, Germany; 4 Gesundheitsamt Köln, Cologne, Germany; 5 Institute of Hygiene, University Hospital Muenster, Münster, Germany; 6 Federal Institute of Public Health (BIÖG), Cologne, Germany

**Keywords:** COVID-19, SARS-CoV-2, infection contexts, infectious disease, nonpharmaceutical interventions

## Abstract

**Objectives:**

Infectious disease outbreaks, most recently the COVID-19 pandemic, have broad societal impacts and may require the introduction of costly non-pharmaceutical interventions (NPIs). To minimize these burdens, the identification of infection contexts is paramount: knowing where infections occur allows the determination of high-risk environments, the identification of outbreak sources, and finally the introduction of targeted, and thus cost-efficient NPIs.

**Methods:**

Starting in February 2020, the Cologne public health department interviewed all PCR-confirmed COVID-19 cases residing in Cologne regarding their probable infection context. The collected individual-level data was entered into an in-house-developed software. From these interviews, aggregated case numbers for the different infection contexts are available at the daily level. The manuscript provides an explorative analysis of the aggregated data.

**Results:**

During 2021, within-one’s-own-household was the dominant known infection context in Cologne. Following within-own-household-infections, the contributions differed over time, with notable contributions of shared accommodations (specifically nursing homes) early in the year, large contributions of disease import during the summer of 2021, and increasing contributions of family (different household) and leisure miscellaneous over the course of 2021.

**Conclusion:**

The analysis reveals that the combination of state-mandated NPIs and voluntary behavior adaptations strongly influenced the regional spreading dynamics and managed to limit infections in critical contexts, such as nursing homes.

## Zusammenfassung

**Zielsetzung:**

Epidemien und Pandemien, zuletzt die COVID-19-Pandemie, haben weitreichende gesellschaftliche Auswirkungen und können die Einführung kostenintensiver nichtpharmazeutischer Interventionen (NPIs) erfordern. Um diese Kosten zu minimieren, ist die Identifikation von Infektionskontexten von essenzieller Bedeutung, denn, zu wissen, wo Infektionen auftreten, ermöglicht die Bestimmung von Hochrisikoumgebungen, die Identifizierung von Ausbruchsquellen und die Einführung gezielter und damit kosteneffizienter NPIs.

**Methode:**

Im Februar 2020 begann das Gesundheitsamt Köln alle PCR-bestätigten COVID-19-Fälle mit Wohnsitz in Köln zu ihrem wahrscheinlichen Infektionskontext zu befragen. Die gesammelten Individualdaten wurden in eine im Gesundheitsamt entwickelte Software eingepflegt. Aus diesen Befragungen liegen aggregierte Fallzahlen für die verschiedenen Infektionskontexte auf Tagesebene vor. Das Manuskript stellt die explorative Analyse der aggregierten Daten vor.

**Ergebnisse:**

Im Jahr 2021 war der eigene Haushalt der dominierende bekannte Infektionskontext in Köln. Nach Infektionen im eigenen Haushalt variierten die Beiträge über die Zeit: Zu Beginn des Jahres 2021 erfolgte ein erheblicher Anteil der registrierten Fälle in Gemeinschaftsunterkünften (insbesondere Pflegeheimen), im Sommer 2021 gab es eine erhöhte Zahl von Infektionsimporten durch Reiserückkehrer/innen, und im Verlauf des Jahres trugen Sozialkontakte zu Familien anderer Haushalte und sonstige Sozialkontakte zunehmend zu den Fallzahlen bei.

**Fazit:**

Die Analyse zeigt, dass die Kombination aus staatlich verordneten NPIs und freiwilligen Verhaltensanpassungen die regionale Ausbreitungsdynamik stark beeinflusste, und es schaffte, Infektionen in kritischen Kontexten, wie etwa Pflegeeinrichtungen, zu reduzieren.

## Introduction

Outbreaks of infectious diseases, most recently and notably the COVID-19 pandemic, often have broad societal impacts, ranging from their effect on the healthcare system on the one hand, to social, psychological, and economic consequences on the other [1]. Infectious disease outbreaks may burden the healthcare system such that hospitals may become saturated with patients, exceeding the bed, equipment, and intensive care unit capacity [2], [3]. Furthermore, quarantine may isolate individuals, weaken social connections, and increase individuals’ anxiety and feeling of uncertainty. During such outbreaks, individuals are often plagued by fear of infection and potential trauma from loss of loved ones or personal health threats on the one hand, and increased stress due to economic uncertainties on the other hand [4]. Finally, on an economic level, infectious disease outbreaks may reduce workforce productivity, cause job losses and income instability, affect and interrupt supply chains, lead to increased healthcare and social support expenses and potentially end in long-term economic recession or slowdown [5].

The impact of disease outbreaks is highly comprehensive, and keeping societal burdens during such an outbreak to a minimum is very important. Bearing this in mind, identifying specific infection contexts and their contributions to overall spread is paramount for effective disease mitigation and supports minimization efforts in several ways:

1. Identification of infection contexts allows public health officials to identify high-risk environments, revealing the environments and settings in which the disease is most likely to spread. Such high-risk environments might include workplace clusters, healthcare facilities, schools, public transport, or social gatherings [6], [7].
2. The identification of the probable infection context aids the identification of the potential source of the outbreaks. It increases understanding of how the disease is spreading and in turn, allows public health officials to track connections between different infection clusters.
3. Knowledge of specific infection locations enables precise, targeted interventions that can effectively control transmission while minimizing the social, psychological, and economic burdens associated with general lockdowns. Examples may include the implementation of localized quarantine measures, the provision of targeted education and prevention efforts, and if necessary, the closure of high-risk activity types and locations.

During an outbreak investigation, case interviews are one valuable qualitative research method to collect detailed data on the circumstances and potential sources of disease transmission. By conducting in-depth, structured interviews with index cases, public health authorities and/or researchers can systematically gather data on recent contacts, locations visited, potential exposures, and specific events or interactions that might have contributed to the infection [8], [9], [10]. Such case interviews were conducted by the Cologne public health department, starting with the registration of the first COVID-19 case in the city of Cologne in February 2020. In these interviews, the Cologne health department collected, among other things, information on infection contexts.

This study analyzed the data collected by the Cologne public health department. The contributions of different infection contexts to the overall infection dynamics were quantified, and how these contributions changed over the course of 2021 was examined. The influence of within-household infections is highlighted, and the increasing influence of disease import following the school summer holidays of 2021 as well as the mitigation effect of targeted pharmaceutical and nonpharmaceutical interventions are discussed. The findings are compared to international research results and highlight how the identification of COVID-19 infection contexts is not only relevant in hindsight for the COVID-19 pandemic but may also aid disease mitigation during potential future outbreaks.

## Method

### Data collection

Starting with the first COVID-19 case reported in the city of Cologne in February 2020, the Cologne public health department (CPHD) contacted all index cases residing in Cologne via telephone. The index cases were interviewed regarding their probable infection context (Table 1 (Tab. 1)). If applicable, the CPHD collected relevant information surrounding the index case’s most recent travel. In this process, the CPHD furthermore collected information on all in-person contacts from the last two days prior to the positive test result or before symptom onset, along with the index case’s symptoms, comorbidities, risk factors, and vaccination status. The collected data were entered into the in-house developed software system DiKoMa (Digital Contact Person Management) [11]. Then, employees from the CPHD either called or e-mailed contacts of the index case to provide them access to their own digital user profile. In their user profile, the index case and their contacts were given the opportunity to keep an online symptom diary. Furthermore, with the help of the DiKoMa software and in line with the contemporaneous regulations of the federal government of the federal state of North Rhine-Westphalia (NRW, the federal state in which Cologne is located), the orders for isolation and quarantine were automatically sent out.

In November 2021, in response to the significant increase in cases, the CPHD began streamlining processes: Initially, not all contacts were called by phone; later, not all index cases were called. Then, the surge of cases during the initial Omicron wave in late 2021 required further adjustments and prompted the CPHD to prioritize contacting vulnerable groups (e.g., persons over the age of 70, persons residing in nursing or community care facilities). To further decrease the department’s workload, citizens, after providing an e-mail address, were given the opportunity to self-register in DiKoMa and create a user profile. From December 2021 onwards, self-entry of previous illnesses and vaccination status, and from February 2022 onwards, self-entry of infection context was possible. This self-entering process facilitated data collection and allowed the department to assess individual risk for severe disease progression during high-incidence periods.

### Data analysis

This analysis focuses on the infection contexts identified via the telephone interviews and the self-entered data from 2021. Other COVID-19 data collected by the CPHD are not considered. The analysis was performed in R [12], and relies on the tidyverse [13]. While Broichhaus et al. [14] used the same data source, their focus lay on 2020, and they did not look at developments over time.

## Results

### Cases by category

Throughout 2021, there were a relatively large number of infections for which the infection context is *unknown* (Figure 1A (Fig. 1); see Figure 1B (Fig. 1) for the 7-day-incidence/100,000 in Cologne). The second-largest observed infection context, after unknown, is own household. Within-own-household infections are critical for the mitigation of infectious diseases: On the one hand, if spread between households is completely suppressed, then the only secondary cases that might stem from an index case are within-own-household infections and the disease will eventually die out [15], [16]. On the other hand, if the effective R-value in private households is larger than one, then any small probability to spread between households may lead to exponential spreading. To target spread within households, the government may introduce nonpharmaceutical interventions (NPIs), such as quarantine hotels for infected persons and their contacts, e.g., as practiced during the COVID-19 pandemic in Italy and the US [17], [18]. However, throughout the COVID-19 pandemic, German decision-makers opted against implementing within-household measures, which proved to be justified: because of a secondary infection risk in households sufficiently far below one [19], they were never necessary. Nevertheless, the permissible band of acceptable infections outside households may be small [15].

The next observation was that the Delta summer wave, starting in July 2021, was initiated by disease import. This import was followed by a large number of household infections as well as increasing infection numbers in all remaining contexts. This summer wave subsided in late September 2021, following the cessation of disease import with the end of summer vacation.

### Cases as shares

Despite providing some insight, as long as reducing within-household infections is not considered for disease mitigation, showing within-household infections as the leading infection is not constructive: It can even be counter-productive, since the misleading perception that most infections happen in households may arise, which would make any disease mitigation strategy meaningless [20]. To address this drawback, we removed the infections for which the infection context is unknown and plotted the shares of the known infection contexts over time (Figure 2 (Fig. 2)).

Figure 2 (Fig. 2) is, however, still misleading in what it communicates. For example, the strong share of disease import during summer 2021 pushed down the share of within-household infections, consequentially making them seeming less important, despite the fact that their contribution to the infection dynamics remained the same.

### Infections relative to within-household infections

For the aforementioned reasons, we divided all case numbers by the number of cases at home for the same time period (Figure 3 (Fig. 3)).

At the beginning of 2021, there were relatively few infections outside one's own household; the population largely complied with the restrictive regime and/or voluntarily reduced contacts and activities outside their own household (see supplementary Fig. S2 and supplement section 5 in Attachment 1). The main infections stemmed from shared accommodations, including (not shown in Figure 3 (Fig. 3)) nursing homes. These, however, subsided fairly quickly because these institutions were prioritized in the vaccination campaign [21]. The work environment was a significant infection context in early 2021, but its prevalence gradually declined following the implementation of working-from-home policies [22]. The remaining infections stem, in equal parts, from family (different household) and leisure miscellaneous. Schools and daycare were mostly closed during the first half of 2021.

Starting in June 2021, a change in the contributions of the different infection contexts can be observed: By then, the majority of the adult population was vaccinated (see supplementary Fig. S1 in Attachment 1) and restrictions were relaxed (see supplement section 5 in Attachment 1). Furthermore, COVID-19 infection dynamics in Germany were generally assumed to be sub-critical (R<1) because of the summer effect. Thus, during the summer of 2021, an increased number of infections stemmed from contacts with friends, but the truly striking feature is the enormous number of disease imports – more than twice as many cases as generated within households. However, these disease import numbers are probably skewed, as one person of the household may have become infected while traveling, then (while still traveling) carried the infection into the family and finally the whole infected family returned. Consequently, all infected family members were counted as *disease import*, while all but one could also be counted as within-household infections. Unfortunately, the data do not allow this distinction to be made.

In addition, in the summer of 2021, infections contracted in schools (educational setting) increased, due to the (then) low vaccination rate among pupils compared to adults (see supplementary Fig. S1 in Attachment 1). The possible contribution of schools during the period before the population was vaccinated, i.e., before summer 2021, cannot be assessed from this data, since schools were largely closed (see supplement section 1 in Attachment 1). However, it is apparent that schools, even with the suppression regimens operating in fall 2021 (rapid antigen testing three times per week in secondary schools, pool PCR testing twice per week in elementary schools, masks during classes until mid-October, improved air quality, see supplement section 1 in Attachment 1), had a sizable impact once opened. The local minimum in October is a consequence of two weeks of school vacation. After that, mask mandates at schools were lifted for a couple of weeks, and universities reopened. 

In November 2021, contributions of leisure miscellaneous and educational setting showed a brief increase, before gradually declining. This is the period of the growing second Delta wave, when the population again reduced their mobility and contacts (see supplementary Fig. S2 in Attachment 1). The miscellaneous peak in November is a consequence of the Cologne carnival, which has a large event on the 11^th^ of November, and where despite attempts to have festivities outdoors, much activity happened indoors. Finally, at the end of December, there is a Christmas peak, consisting of disease import, family (different household), and leisure miscellaneous.

## Discussion

Our analysis supports the hypothesis that the introduction of (non-)pharmaceutical interventions, both by the national and federal government, had a strong influence on the regional COVID-19 spreading dynamics. The vulnerable contexts of nursing homes and hospitals were targeted and thereby defused by vaccinations early; voluntary and state-mandated contact reductions reduced infections in spring 2021. Our analysis also shows that disease import has an enormous influence. The only reason why Germany did not experience exponential growth of case number in July/August 2021 was that the overall R-value, because of the recent vaccinations and the summer effect, was below one, and thus the disease import did not trigger intrinsic exponential growth. 

It is important to note that this disease import does not only concern new variants, but any travel activity where the probability to become infected is larger than at home. This can, for example, concern a stay in an area with a higher incidence than at home, a travel mode with a high probability to become infected while en route, or more indoors visits than at home even when incidences are similar.

A comparison with data from the canton of Zurich is revealing, because the canton is the only other location that provided data on infection contexts in sufficiently similar categories and over time [23]. When plotting either the infection numbers or the infection shares over time, the same issue arises as for the city of Cologne; the data varies wildly (see supplementary Fig. S3 and Fig. S4 in Attachment 1). However, when dividing the number of infections for each infection context by the within-one’s-own-household infections, the results fluctuate even less than for the Cologne data (Figure 4 (Fig. 4)). Furthermore, the overall trend is similar: Small quotients until the summer, then strong disease import and large fluctuations, and considerably more out-of-home infections after the summer.

For other locations, information on infection contexts is only available for a single point in time (for details see supplementary material in Attachment 1), which limits the comparability to the Cologne data. Furthermore, even when wanting to compare the prevalence of different infection contexts across nations at single points in time, one must be extremely careful as different countries introduced nonpharmaceutical interventions that targeted different activity types. Still, we note that, once large events are prohibited and care facilities are targeted via vaccinations and/or visiting bans, the community transmission consists of work contexts, educational contexts, non-household family contexts, and non-family leisure contexts in approximately equal shares.

## Limitations

Unfortunately, this analysis was only possible from 2021 onwards. In 2020, the Cologne health department did not differentiate the infection contexts own household, family (different household), and leisure miscellaneous, but collectively called them social contacts. Additionally, with rising infection numbers in late 2021, the public Cologne health department was unable to reach out to all contact persons and later all index cases. Hence, underreporting and incomplete data must be assumed from late November 2021 onwards and consequently, this analysis focuses on 2021 only.

Another limitation is the index cases’ compliance in case interviews: An index case may be influenced by fear of official prosecution when admitting to the disregard of the ordered measures (for example when admitting to attending a prohibited indoor gathering) and/or a general distrust of public authorities [24]. They may be scared of social consequences when naming social contacts as contact persons and/or possible infection sources, as this may result in ordered quarantine for the contact(s). Individuals may furthermore be unable to provide adequate information on their infection context and/or contact history due to a language barrier or a lack of understanding of the circulating virus. Finally, it shall be noted that the collected data is based on human memory, which has its failabilities [25].

## Conclusion

For future potential epidemic outbreaks, we conclude: The determination of infection contexts from case interviews is a powerful tool to identify high-risk environments and potential outbreak sources to then determine mitigation strategies if TTI fails. In such case interviews, it is paramount to have “within-one's-own-household” as a separate category when determining infection contexts. This is important since social contacts outside one’s own household are easier to reduce and cut than contacts inside one’s household. The division of the infection numbers of all other contexts by the number of infections at home allows a precise assessment of the relative contributions of each infection context, independent from changing incidences or changing regimes. If, in addition, the secondary attack rate at home is known, then one can even determine the permissible share of other infections. This allows to fine-tune suppression measures even when little is known about the exact transmission routes of the disease.

To facilitate the determination of infection contexts, public health departments should have software that allows infected people to register and self-enter the infection contexts. This allows the health departments to concentrate on persons unable to do this by themselves, thus economizing scarce resources.

It will not necessarily be the case that a future pandemic will follow the same (largely aerosol-based) transmission route. In consequence, health departments need to be ready to accept other problematic infection contexts than those for COVID-19.

## Notes

### Authors’ ORCIDs

- Hurraß J: https://orcid.org/0000-0002-9512-9338
- Paltra S: https://orcid.org/0000-0002-4935-8586
- Müller S: https://orcid.org/0000-0002-3103-8955
- Kossow A: https://orcid.org/0000-0002-4648-4851
- Nagel K: https://orcid.org/0000-0003-2775-6898

### Ethical approval 

Ethical approval is not required, as all analyzed data is fully and irreversibly anonymized.

### Funding

The data was collected by the public health authority of Cologne (Gesundheitsamt Köln) as part of their efforts to manage COVID-19. The work on the paper was funded by the Ministry of research and education (BMBF) Germany (grant numbers 031L0300D, 031L0302A), Gesundheitsamt Köln, and TU Berlin.

### Acknowledgments

We thank Rafael Mikolajczyk for insightful discussion and helpful feedback.

### Competing interests

The authors declare that they have no competing interests.

## Supplementary Material

Supplementary materials

## Figures and Tables

**Table 1:**
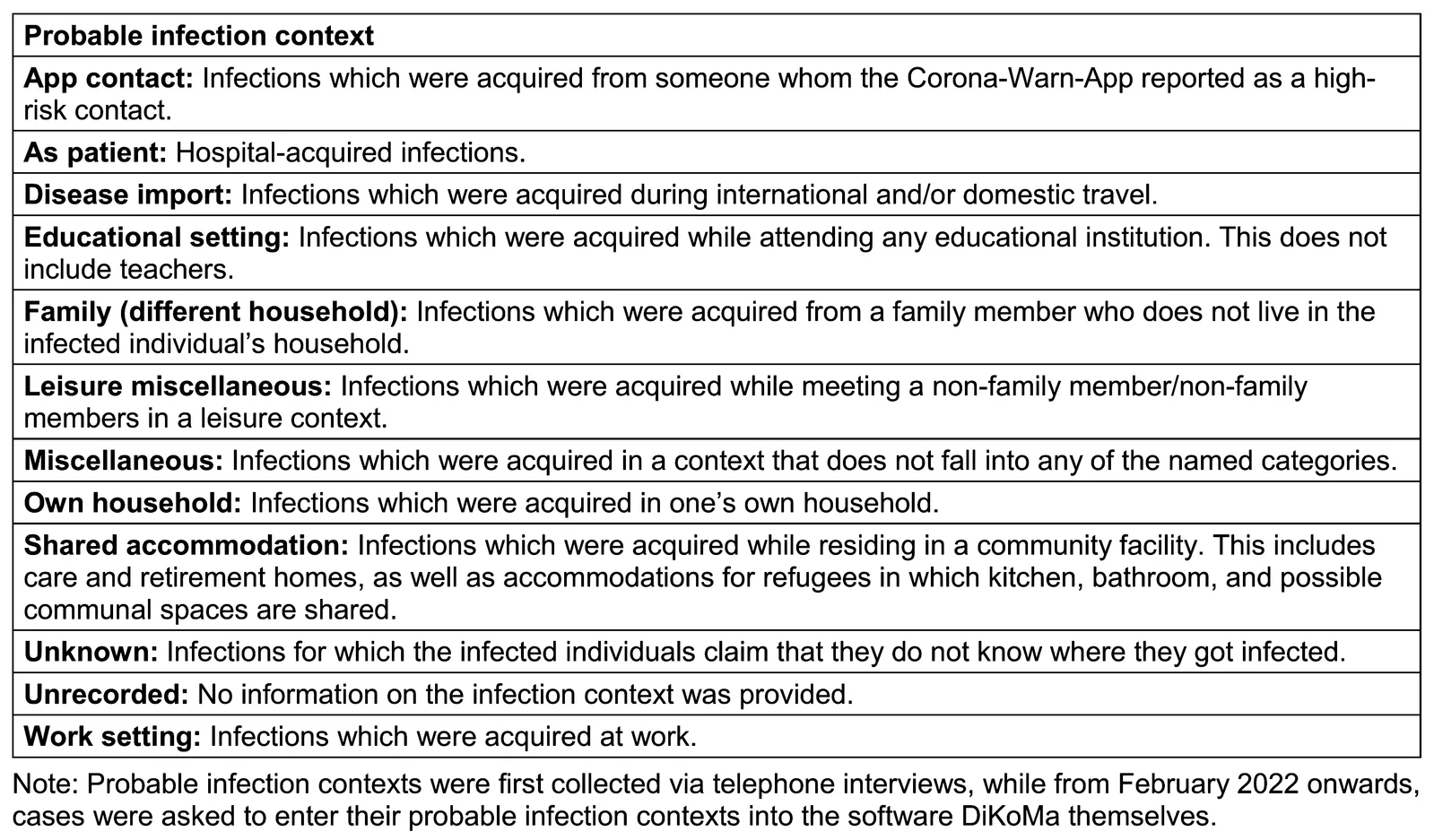
COVID-19 infection contexts as recorded by the CPHD

**Figure 1:**
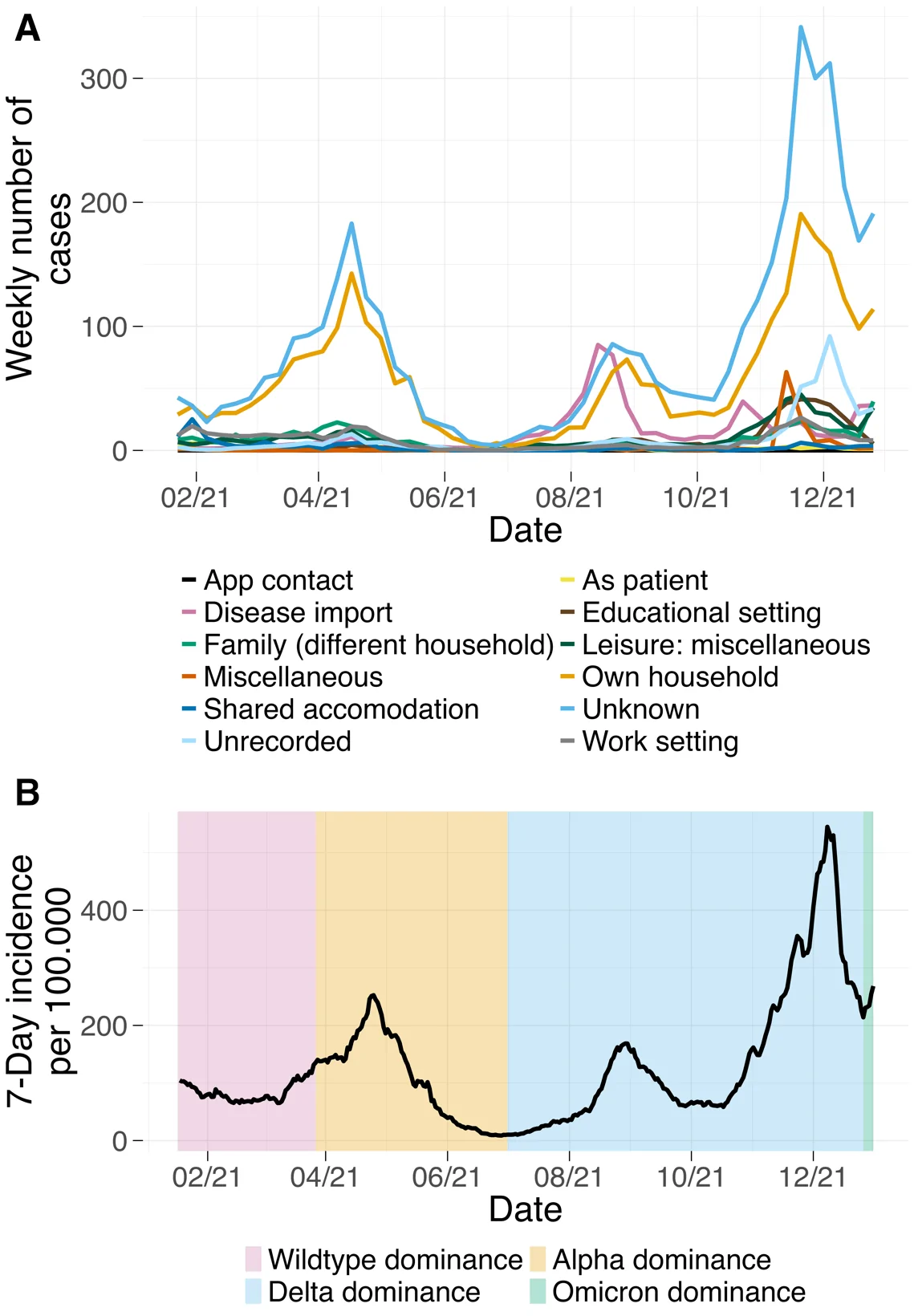
A. Weekly number of cases per infection context in the city of Cologne. B. Daily 7-day-incidence/100,000 for the city of Cologne (color coded with the virus strain dominant at that time). The graph depicts the remainder of the wildtype winter wave (pink), the Alpha wave (orange), the first Delta wave caused by disease import (see Figure 1A, Figure 2), the second Delta wave peaking in November 2021 (blue), and the onset of the Omicron wave (green). Data source: RKI VOC and weekly reports for virus strain dominance in NRW [22]

**Figure 2:**
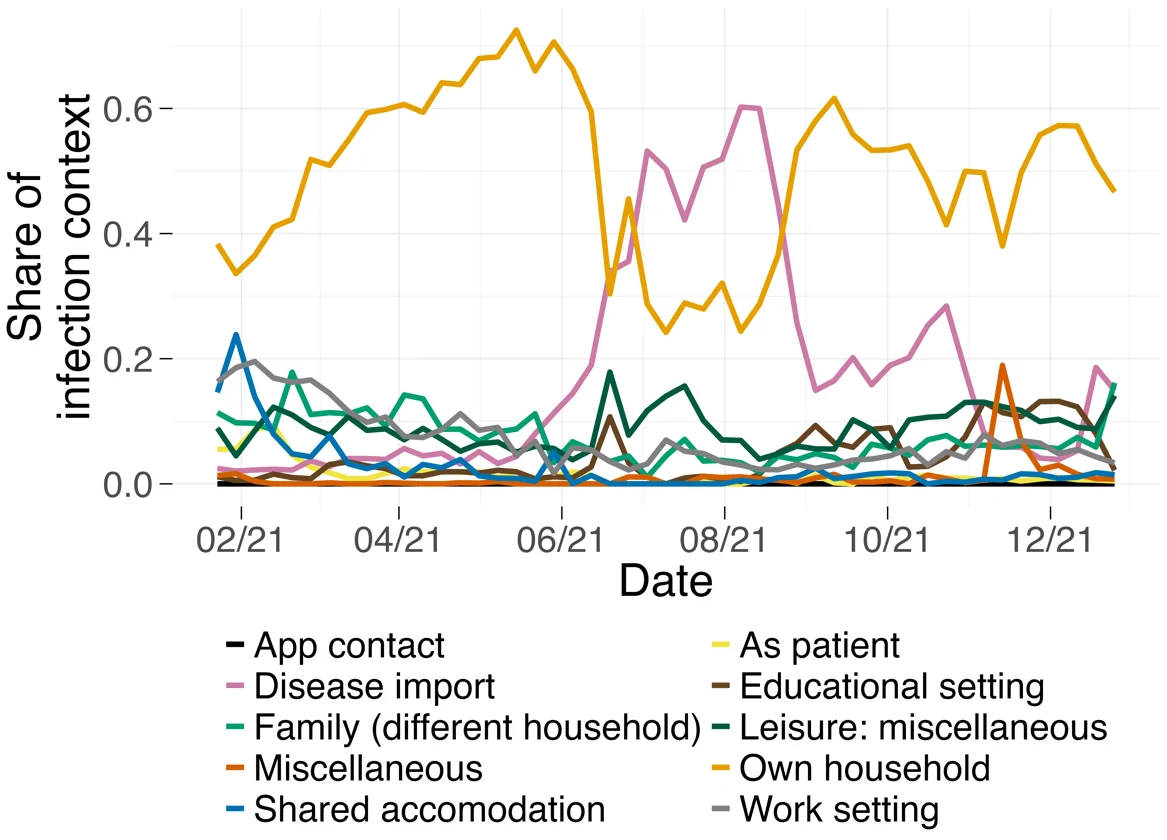
Shares of different infection contexts relative to the sum of all cases with an identified infection context

**Figure 3:**
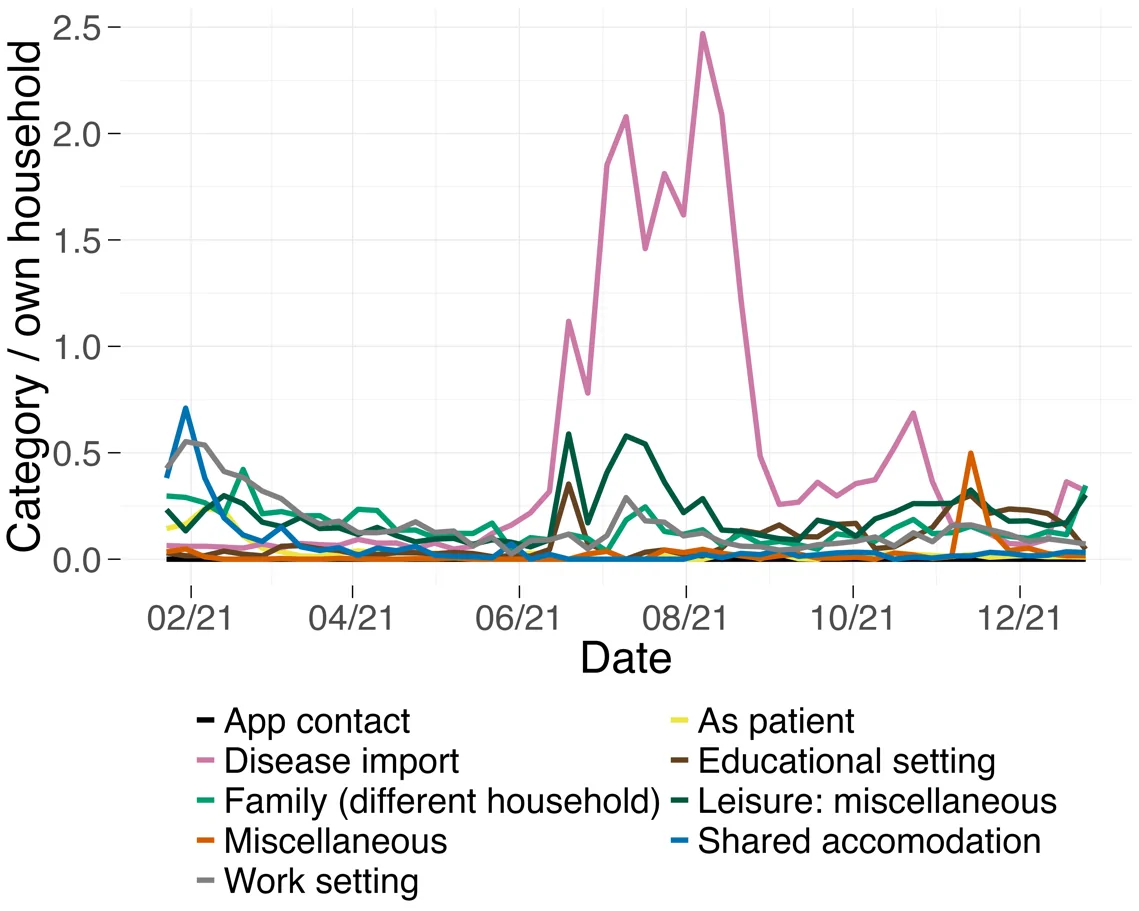
Weekly number of cases for each considered category divided by weekly number of cases within-own-household for the city of Cologne

**Figure 4:**
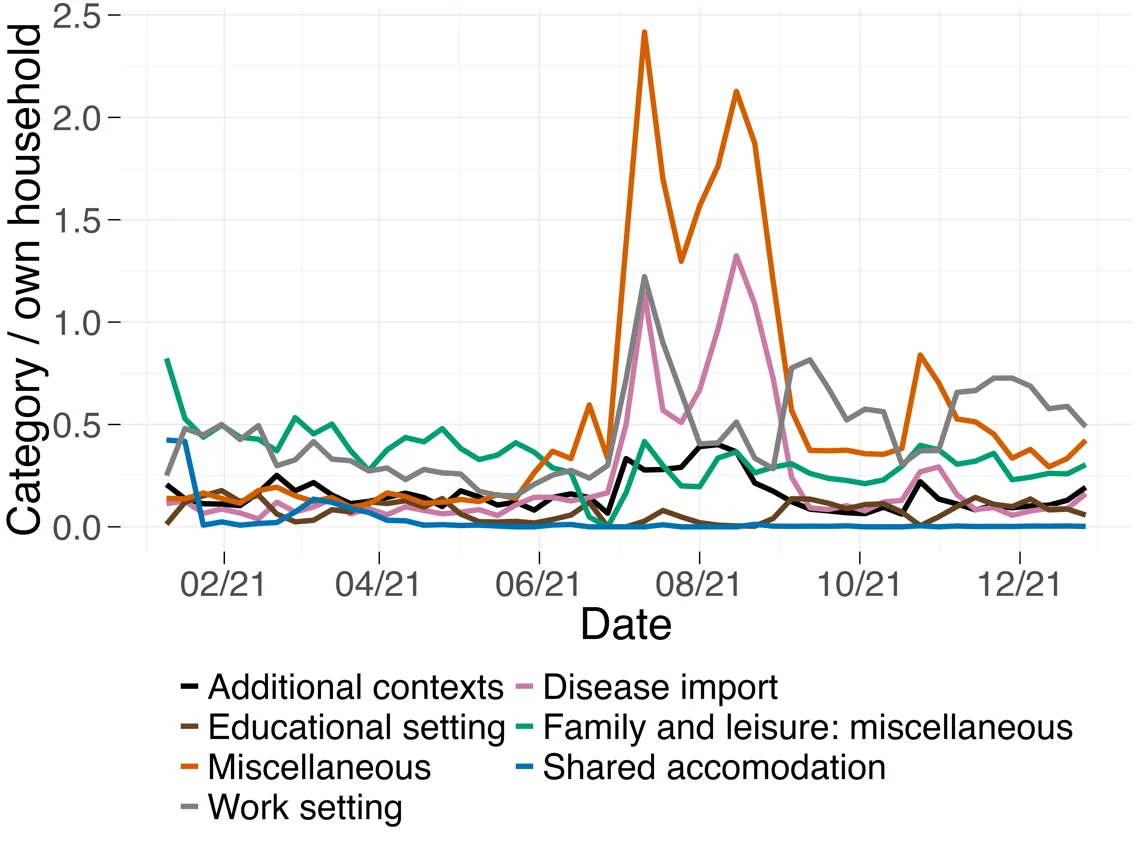
Weekly number of cases for each considered category divided by weekly number of cases within-own-household for the Canton of Zurich
