# Supplementary material for: Identification of key COVID-19 infection contexts over time and the importance of within household infections
Source: GMS Hyg Infect Control. 2026 Sep 15;21:Doc69. doi: 10.3205/dgkh000678 (PMC13687556; doi:10.3205/dgkh000678)
Supplement: Supplementary file 1 [file HIC-21-69-s-001.pdf]

Supplementary materials

1. Timeline of (partial) school closures/measures in schools during 2021

The information depicted here was collected from the contemporaneous Coronabetreuungsverordnungen of NRW [1].

12/23/2020 - 01/06/2021: Christmas vacation, schools are closed.
01/07 - 02/21: School closures, remote classes offered for all pupils.
02/22 - 03/14: Hybrid lessons ("Wechselunterricht") are permitted at primary schools and schools for children with disabilities. When enacted, the class is divided into two groups: one group may be taught in-person at school while the other must be conducted remotely from home. Additionally, after school care is provided for pupils from first through sixth grade, as well as for pupils with disabilities of all grades whose parents are unable to supervise them after school. Finally, graduating classes may be entirely taught in-person with no compulsory class division implemented. Medical/FFP2 masks must be worn indoors.
03/15 - 03/28: Remaining students may now be taught via "Wechselunterricht" with no additional changes for primary schools, schools for children with disabilities and graduating classes.
03/29 - 04/11: Easter vacation, schools are closed.
04/12 - 04/18: Additional school closures.
04/19 - 05/30: All grades, except graduating classes, continue the hybrid concept.
05/10 : PCR pool tests ("lollipop tests") began implementation at primary schools and schools for children with disabilities twice a week (on Mondays and Wednesdays); Rapid antigen tests at secondary schools began implementation simultaneously.
05/31 - 07/02: As long as a county's or independent city's incidence remains below 100 for at least three days in a row, all students may be taught in-person at school. If the incidence passes 100, schools must return to hybrid lessons; if the incidence passes 165, schools must return to remote learning.
07/05 - 08/17: Summer holidays.
08/16: STIKO now recommends the vaccine for 12-17 year old adolescents.
09/20: Frequency of rapid antigen tests is increased to three times a week (Monday, Wednesday, Friday); vaccinated students do not have to get tested.
10/11 - 10/23: Fall holidays.
12/02: Re-introduction of mask mandates at students' desks.
12/09: STIKO now recommends the vaccine for 5-11 year old children with underlying comorbidities.
12/24/2021 - 01/08/2022: Christmas vacation.

2. Vaccination rate in Cologne

The depicted vaccination rates are our own calculations based on the official vaccination counts provided by RKI, divided by the population numbers by Statistisches Bundesamt [2, 3].

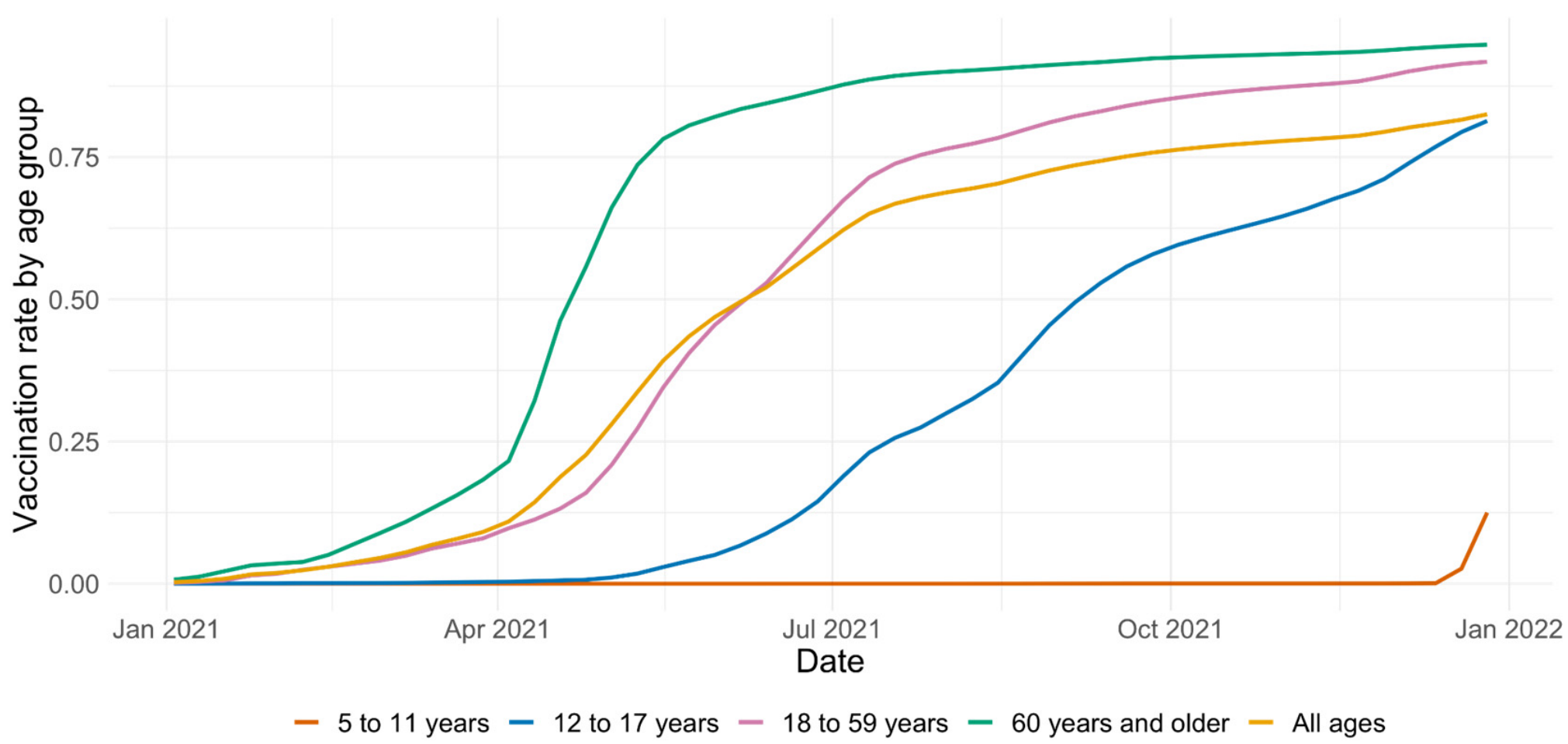

Figure S1: Vaccination rates in Cologne per age group. Due to Germany's priority-based vaccination system, people aged 60 and older (following healthcare workers) received vaccines first, leading to a sequential increase in vaccination rates starting with older age groups and gradually extending to younger populations. The late increase of vaccination rate of 5 to 11-year-old children is due to the late recommendation of the vaccine for this age group and then only for children with comorbidities.

3. Out-of-home duration (Cologne)

The amount of time (in hours) an average person in Cologne spent outside their home per day is depicted in Figure S2.

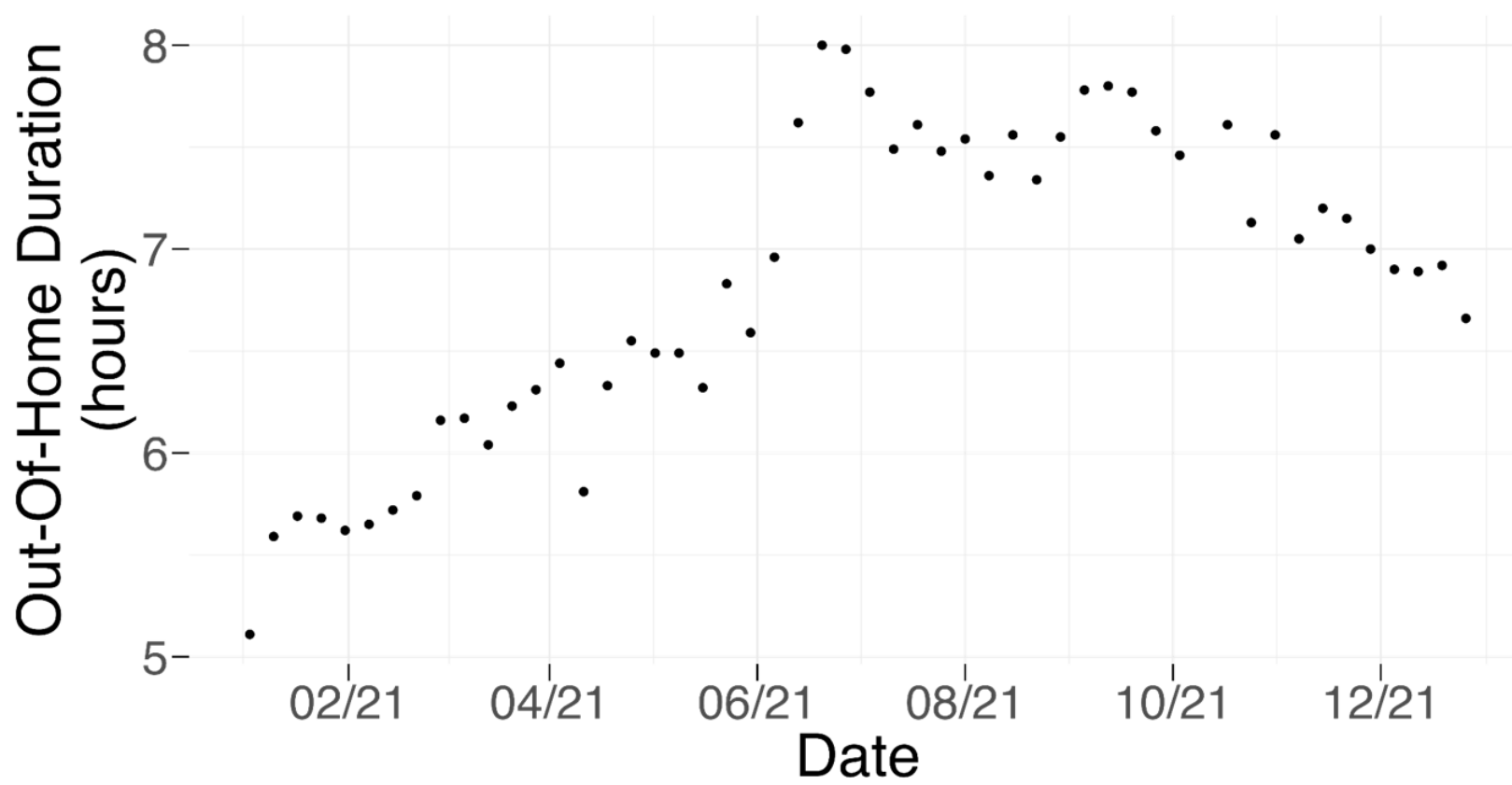

Figure S2: Daily out of home duration per person (in hours) for 2021 for the city of Cologne. The global minimum occurs in early 2021, followed by a slow but steady increase until June 2021. Maximum values are reached during the summer of 2021 with a small valley that may be attributed to the summer vacation. Out-of-home durations decrease again from mid-September onwards.

4. Comparison with other data sets

As noted in the Results section of the main paper, we performed an analogous analysis for the publicly available datasets of RKI and Canton of Zurich. As said section only mentions the main results of this analysis, the following section shall introduce our approach in detail. This section also gives details on the values of Table 2. As different techniques were applied and different contexts were defined when setting up these data sets, we would like to stress once more that one must be cautious when making comparisons between data sets.

Zurich, Switzerland

For both 2020 and 2021 the contact tracing data of the Gesundheitsdirektion Canton of Zurich is published [4] . For 2020 only cases, for which the infection context is unmistakably identified, are included, while for 2021 also cases, for which the infection context is only suspected, are included. Our analysis uses the sum of the unmistakably identified and suspected cases (see Table S1 for list of possible infection contexts).

Table S1: List of infection contexts as recorded by the public health authorities in Zurich, Switzerland

**Probable infection context (english translation)**
Army / civil defense
Cinema / theatre / concert
Club / bar
Event / party with less than 300 people
Family and leisure: miscellaneous
Hairdresser / massage / similar services
Hotel
International travel
Kindergarten
Educational setting (compulsory schooling)
Medical setting
National travel
Miscellaneous
Nursing / retirement home
Own household
Religious event / funeral
Restaurant
Secondary school / university / further education
Shopping
Societies / associations
Sport (indoors)
Sport (outdoors)
Unrecorded
Work setting

Attachment 1 to: *Hurraß J, Paltra S, Wolff A, Grüne B, Müller S, Kossow A, Nießen J, Nagel K. Identification of key COVID-19 infection contexts over time and the importance of within household infections. GMS Hyg Infect Control. 2026;21:Doc69. DOI: 10.3205/dgkh000678, URN: urn:nbn:de:0183-dgkh0006788*

As these contexts do not exactly match the ones considered by the public health authorities in Cologne and to allow some form of comparability, we combine some of the infection contexts as follows:

- **Disease import** = international travel + national travel
- **Educational setting** = secondary school / university / further education center + mandatory school + kindergarten
- **Additional contexts** = army / civil defense + hotel + club / bar + restaurant + cinema / theatre / concert + event / party < 300 people + religious event / funeral + society / association + medical institution + hairdresser / massage / similar + sport (inside) + sport (outside) + shopping

Additionally, we point out that this data set does not offer the distinction of the Cologne categories *family (different household)* and *leisure miscellaneous*. Instead, these two categories are merged into *family and leisure - miscellaneous*. Plotting these categories as is, one obtains:

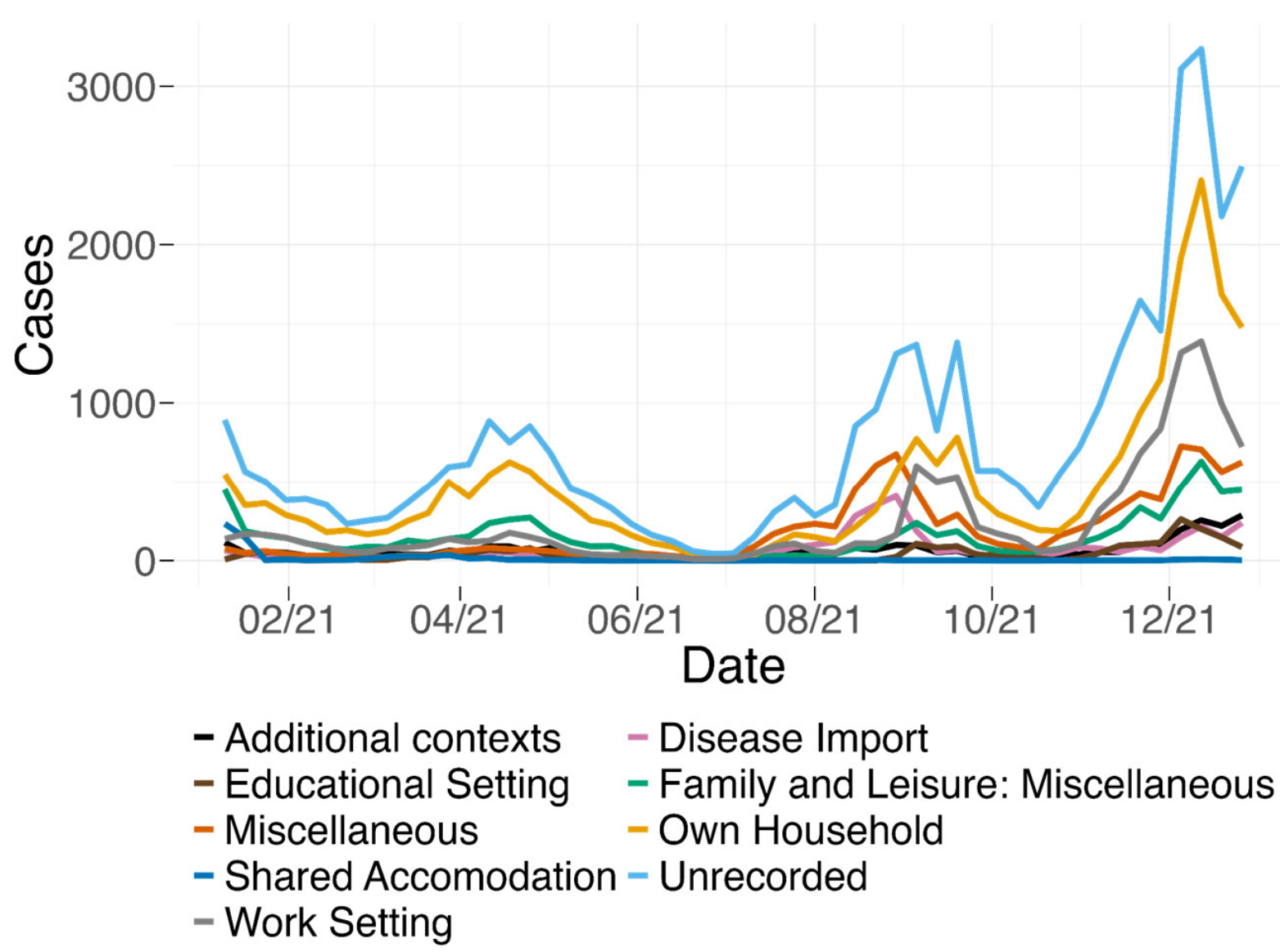

Figure S3: Number of cases per infection contest in the Canton of Zurich over the course of 2021. The three local maxima are due to the alpha variant (April 2021), a late summer wave caused by disease import of the Delta variant (September 2021), and the Delta winter wave (December 2021).

Removing the unrecorded category and plotting the shares, we obtain:

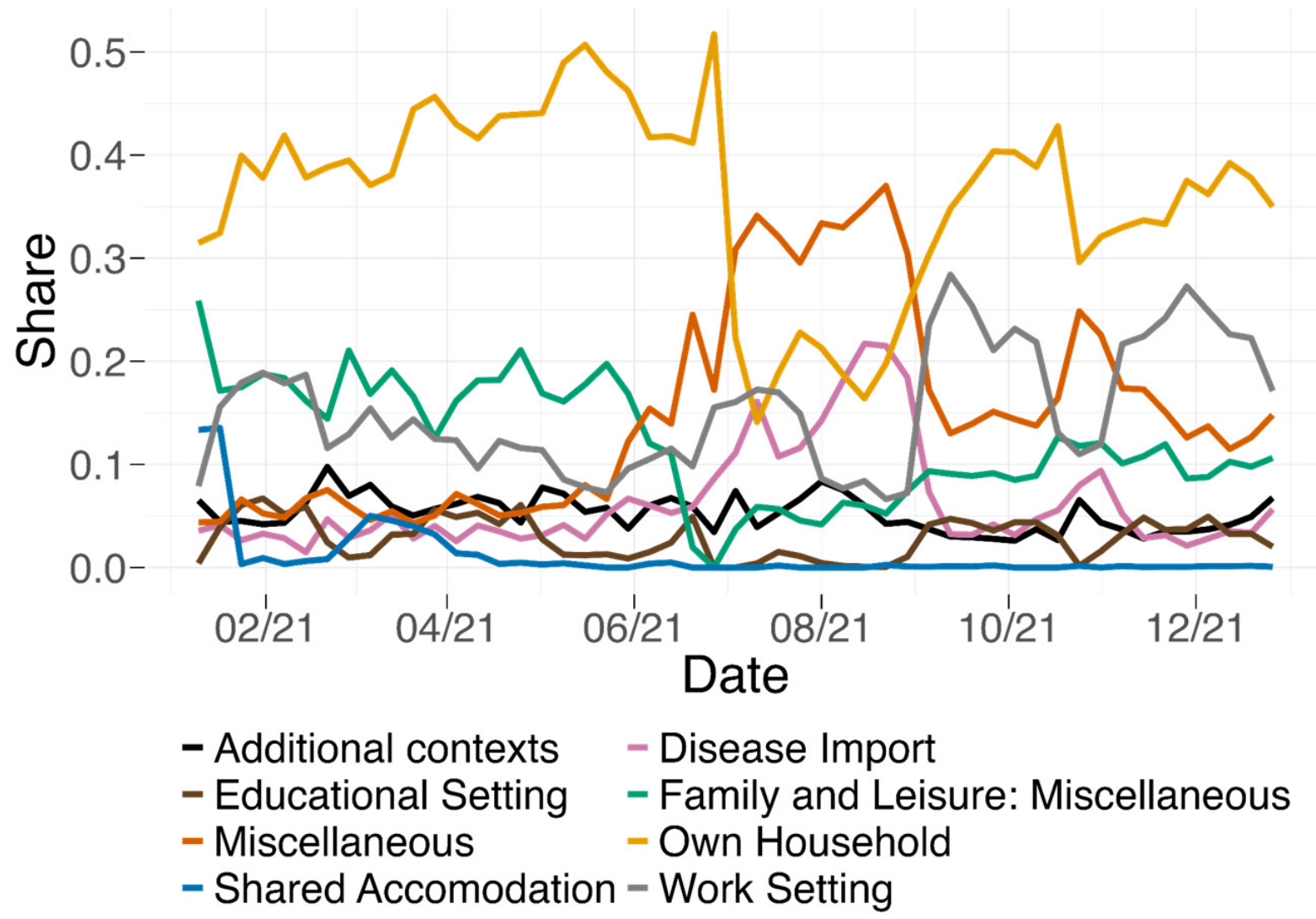

Figure S4: Shares of different infection contexts relative to the sum of all cases. Apart from the summer 2021, where many infections are recorded as d*isease import* and *miscellaneous, own household* is the dominating infection context.

Own household is the dominating infection context, followed by *work setting* and *family and leisure miscellaneous*. Until June of 2021, the share of within-own-household infections is around 40%, while the share of the *family and leisure miscellaneous* infections is around 20% and the share of *work setting* is around 15%. the remaining infection contexts did not contribute considerably. During the summer holidays of 2021 we also see a rise in infections acquired while traveling, contributing up to more than 20% of said cases. After the summer holidays, the contributions from *work setting* and from *family and leisure miscellaneous* become larger, while the *own household* category becomes accordingly smaller.

The more useful plot that divides by the infections at home is shown in the main part of the paper.

Germany

For Germany, national contact tracing data is collected and published by RKI. Considering data until week 29 of 2020, 27% of cases could be matched to at least one outbreak context (here, outbreaks are defined as at least 2 laboratory confirmed, related cases), naming private households, followed by care and nursing homes as the main outbreak settings [5]. In the same study, outbreaks associated with the workplace reached a peak during week 25 and the subsequent weeks, following a large outbreak in the German meat industry. The educational setting only played a minor role, as schools in Germany were totally closed from March 16, 2020 until April 27, 2020 and then partially closed until

the summer holidays. As said study only discusses data until the summer of 2020, we additionally (up until July 13, 2021) considered the daily reports by RKI [6]. Every Tuesday this daily report contained a bar chart depicting the contexts in which outbreaks occurred. From then onwards, RKI did not discuss outbreaks in its reports, but still provided the underlying data on a weekly basis (see Table S2 infection contexts recorded by RKI) [7]. Consequently, we used this data to perform our own analysis. The contexts considered by RKI are:

Table S2: Infection contexts as recorded by RKI. RKI does not differentiate between own household and households of friends, extended family. This limits the comparability to the Cologne data.

**Infection context (english translation)**
Care facility
Doctor’s office
Dorm
Hospital
Kindergarden, child care facility
Leisure
Medical institution
Miscellaneous
Not part of an outbreak
Overnight stay
Private household
Public transport
Refugee accommodation
Rehabilitation centre
Senior daycare
Restaurant
Retirement / nursing home
Educational setting
Senior daycare
Unknown
Work setting

Attachment 1 to: *Hurraß J, Paltra S, Wolff A, Grüne B, Müller S, Kossow A, Nießen J, Nagel K. Identification of key COVID-19 infection contexts over time and the importance of within household infections. GMS Hyg Infect Control. 2026;21:Doc69. DOI: 10.3205/dgkh000678, URN: urn:nbn:de:0183-dgkh0006788*

As these contexts do not match the ones considered by the public health authorities in Cologne, we combined some of the contexts:

- **As Patient** = medical institution + hospital + rehabilitation facility + doctor's office
- **Educational Setting** = school / university + Kindergarten, child care facility
- **Shared Accommodation**[1] = retirement / nursing + refugee accommodation + dorm + Senior daycare

Plotting the shares of the known infection contexts results in the following plot:

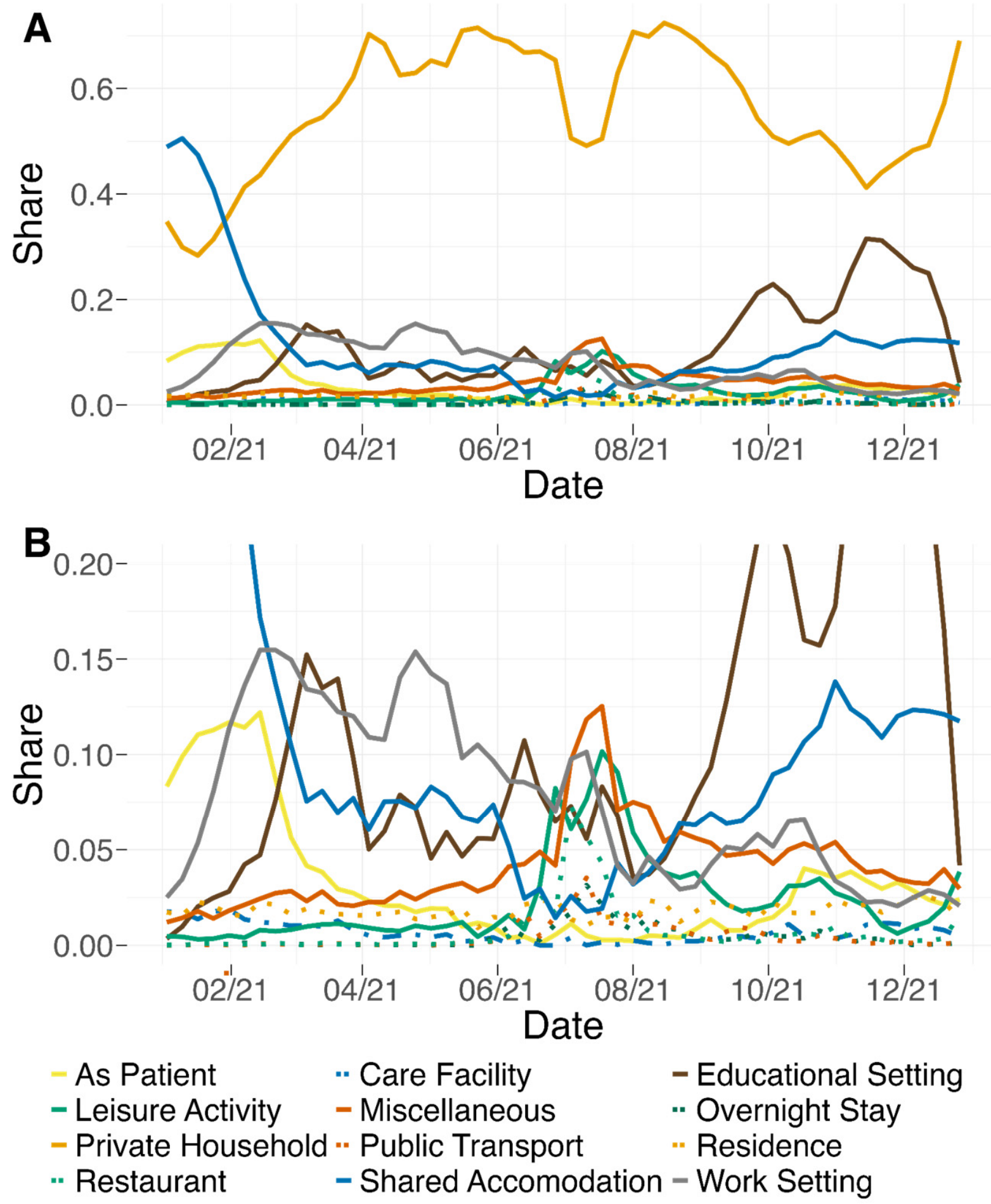

Figure S5: Shares of different infection contexts relative to the sum of all cases that could be attributed to an outbreak and for which the infection context could be identified. The bottom plot shows the same data as the top plot, but on a different scale on the y axis to have more resolution for the infection contexts outside *private households*.

[1] Does not include the context "care facility", as this both includes outbreaks at senior daycare and childcare facilities.

Using the top plot and apart from the very beginning of the year, we identify *private household* as the dominating infection context, whose share never drops below 30%. As RKI does not differentiate between one's own household, the household of family members and the household of friends and acquaintances, our analysis cannot differentiate between these contexts either. At the beginning of the year the share of *shared accomodation* is almost 50%, but as nursing and retirement homes were prioritized during the vaccination campaign, this share soon starts to decrease until it reaches its minimum during the summer and only accounts at around 3%. The additional contexts whose share is up to 15% during the first half of the year are *work setting*, *educational setting*, and *as patient*. During the summer we then see an increase in cases which could be traced back to *leisure activity*, *overnight stays* and *miscellaneous* settings. After the summer holidays, we note a sharp increase in cases traced back to the educational sector, which becomes the second-largest contributor until the end of the year. The remaining infection contexts did not contribute considerably.

Dividing by the number of infections at home is not possible since infections in one's own home and infections in someone else's home are not differentiated. Consequently, comparability to the Cologne data is very limited.

UK

The UK Health Security Agency publishes a weekly influenza and COVID-19 surveillance report in which they depict data on suspected outbreaks (so-called "incidents") and their contexts, of which only a subset will turn into laboratory confirmed outbreaks (again, outbreaks are defined as two or more related cases) [8]. The settings they consider are *care home*, *educational setting*, *food outlet/restaurant*, *hospital*, *prison*, *workplace setting*, and *other*. The data is depicted and described, but no conclusions are drawn. Social settings and most leisure activities are not discussed. Plotting the incidents and their according shares for 2021, we observe that the main contributors in this data set are *care homes* and the *educational setting*, whose shares oscillate and which hence take turns being the dominant setting (Figure S5). Then, from December 2021 onwards *care homes* are the biggest contributor accounting for around 75% of reported incidents. In addition, while in the spring of 2021 work contributes to around a quarter of the reported incidents, its share thereafter drops to around 10% before decreasing to the single-digit percentage range in the fall of 2021. The remaining settings, apart from *other*, all on average have shares in the single-digit percentage range. The category *own household* does not exist in the data. One might expect that they are included under *other*; however, for this we believe the share of other to be too low. One has to assume that within-household infections were simply not considered as incidents.

Attachment 1 to: *Hurraß J, Paltra S, Wolff A, Grüne B, Müller S, Kossow A, Nießen J, Nagel K. Identification of key COVID-19 infection contexts over time and the importance of within household infections. GMS Hyg Infect Control. 2026;21:Doc69. DOI: 10.3205/dgkh000678, URN: urn:nbn:de:0183-dgkh0006788*

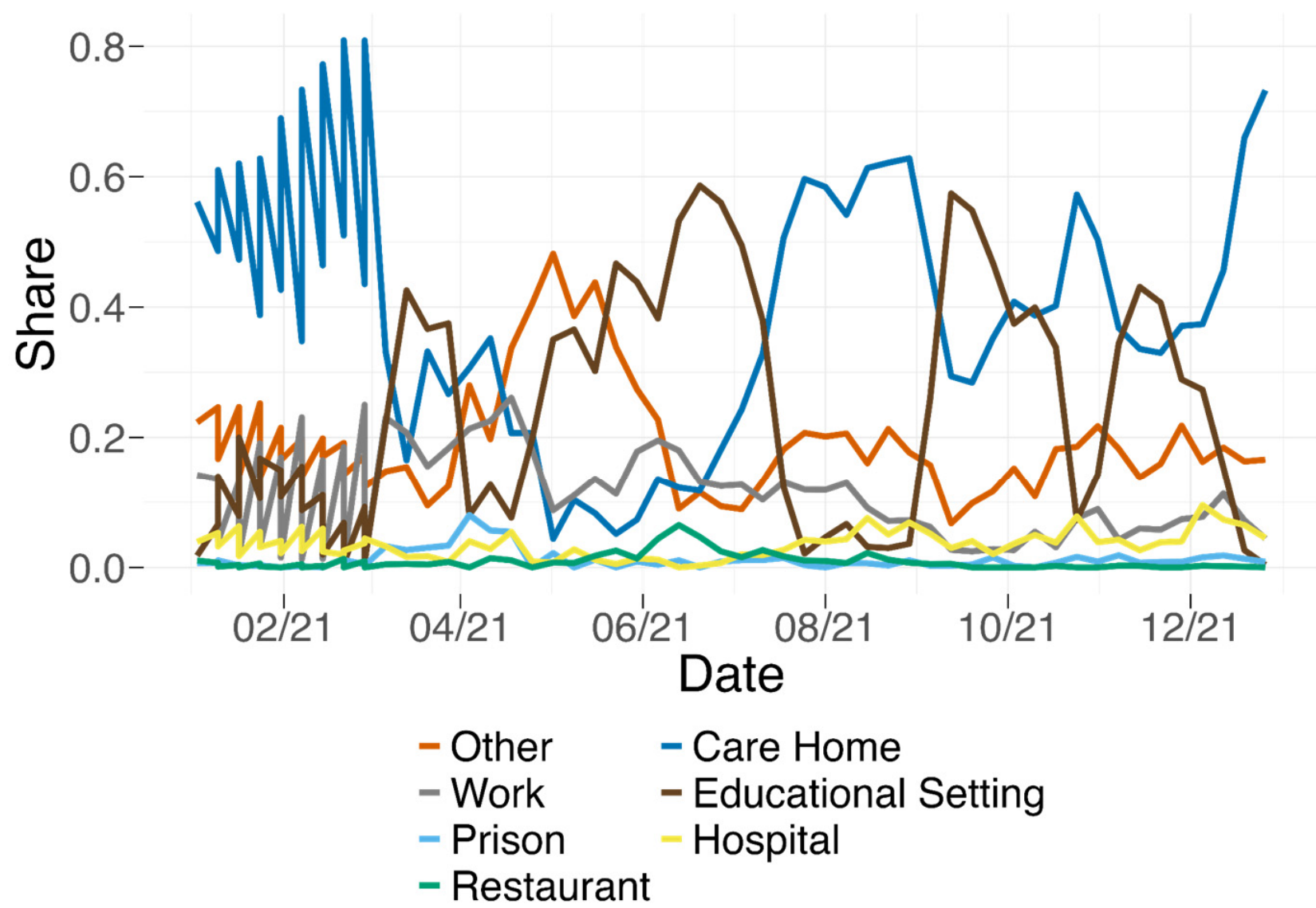

Figure S6: Shares of different infection contexts relative to the sum of all outbreaks in England.

A second source for the contributions of different infection contexts in the UK is a report by the British SAGE group: Here, living with a case, attending family and/or friend gatherings and dining are associated with a higher infection risk than short community encounters [9]. The authors argue that poor ventilation and crowding contribute to outbreaks and consequently identify bars, churches and nightclubs as likely infection contexts. Finally, they note that the largest outbreaks across the world have been reported in long-term care facilities (e.g. nursing and retirement homes), homeless shelters, prisons and low wage workplaces like meat processing plants. They do, however, not perform an analysis over time.

Luxembourg

The Luxembourg Government spends a section on the identification of infection context in its weekly COVID-19 review [10]. In the week of May 2 to May 8, 2022 the source of 26% of the reported cases could not be identified, but 38% of transmissions can linked to the *family circle*, 12% are traced back to *educational settings*, 8% are linked to *leisure activities*, 5% to *work* and 5% to *travel abroad*. As the aforementioned week in 2022 does not overlap with our study period, and as no analysis over time was performed, a comparison to our analyses results was not possible.

South Korea

For the South Korean Gangwon Province, cluster infections (here defined as at least 10 linked cases within a short period of time) were investigated between February 22, 2020 and May 31, 2021 [11]. The considered clusters included more than half (55.7%) of the confirmed cases of that time period. Here, the infection contexts *using (staying in) the same facility*[2] (accounting for 50.7% of cluster infection cases), *cohabiting family members* (23.3%), *social gatherings with acquaintances* (10.8%), *others* (7.0%) and *social gatherings with non-cohabiting family members/relatives* (5.5%) and *unknown* (2.8%) were considered. For *using (staying in) the same facility*, *offices*, *restaurants*, *churches*, *schools*, *medical institutions* and *sports facilities* were the main contributors. For the infection contexts *social gatherings with acquaintances* and *social gatherings with non-cohabiting family members/relatives*, *home* was the leading subcontext, accounting for almost 30% of infections of those two contexts. However, as cluster infections instead of single infections are discussed, the comparability to our analyses is limited.

Japan

Through case interviews possible sources of infection were identified in the early stage of the pandemic in Japan [12]. Here, the focus lay on the identification of cluster (defined as at least 5 cases) contexts. *Healthcare facilities* (30% of clusters), *care facilities of other type* (i.e. nursing homes and day care centers, 16%), *restaurants or bars* (16%), *workplaces* (13%) and *music-related events* (11%) were identified as the most common contexts, while *gymnasiums*, *ceremonial functions* and *transport-related incidents in airplanes* were also considered. A plot over time is provided, but only until April 2020, limiting the comparability to our analyses.

Spain

The national COVID-19 outbreak monitoring group considers outbreaks notified to the national level in Spain during the beginning of summer of 2020 [13]. They define outbreaks as three or more cases and discuss 673 of said outbreaks consisting of 8,390 cases. They identify *social settings* (responsible for 30.6% of outbreaks) and *occupational settings* (responsible for 21.7% of outbreaks) to be the two main settings in which outbreaks originated. *Social settings* consist of five different settings, namely *family/friends reunion of private party* (17.8%), *leisure facility (restaurant, bar, club …)* (5.2%), *organized event/public space* (4.6%) and *other/not specified* (3.0%). In addition to that, they attribute 9.7% of outbreaks to *family - different households*, 8.8% to *long-term care facility* and 3.0% to

---

[2] Includes bath houses, child care, churches, colleges/universities, day care facilities, day care centers and kindergartens, funeral homes, hobby schools, kitchens, learning centers, lodging, medical institutions, military bases, nightlife businesses, nursing homes, offices (both workers and visitors), public institutions (both workers and visitors), restaurants, schools, service, businesses, shipping, sport facilities, stores, visiting nursing homes.

healthcare facility. They quantify the contributions of different settings over time, but as there exists no overlap between their and our study period, no detailed comparison was performed.

China

Qian et al. discuss the case reports from the local Municipal Health Commission of 320 prefectural cities in China (excluding Hubei Province) in the very first phase of the pandemic between January 4 and February 11, 2020 [14]. Based on common index patients, they identify outbreaks (consisting of three or more cases) for which they consider six infection contexts: *homes*, *transport*, *restaurants and other food venues*, *entertainment venues*, *shopping venues* as well as a *miscellaneous* category. Among the 318 identified outbreaks, 235 outbreaks were associated with only one venue: 188 outbreaks occurred in a *home*, 18 outbreaks occurred on *transport*, 10 occurred at a *restaurant*, 6 occurred at an *entertainment venue*, 7 occurred at a *shopping venue* and the final 6 occurred at a *miscellaneous venue*. Additionally, they consider four relation categories for the infected individuals: *family members*, *family relatives*, *socially connected individuals* (including friendships, acquaintances, jobs) and *socially non-connected individuals*. They do not specify which (if any) of these categories imply that the related individuals share a household; one might speculate that *family members* means that they are living in the same household whereas *family relatives* means that they are not. The 318 outbreaks consist of 129 involving only *family members*, 133 involving *family relatives*, 29 involving *socially connected individuals*, 24 involving *socially non-connected individuals* and only 3 involving multiple relationships.

5. Timeline of introduced measures in 2021 in Cologne

In Germany, during the COVID-19 pandemic, NPIs were introduced on the national, federal state and sometimes city level (see corresponding Coronaschutzverordnung of NRW and national Arbeitsschutzverordnung [1, 15]). The timeline below depicts the combination of these measures for the city of Cologne.

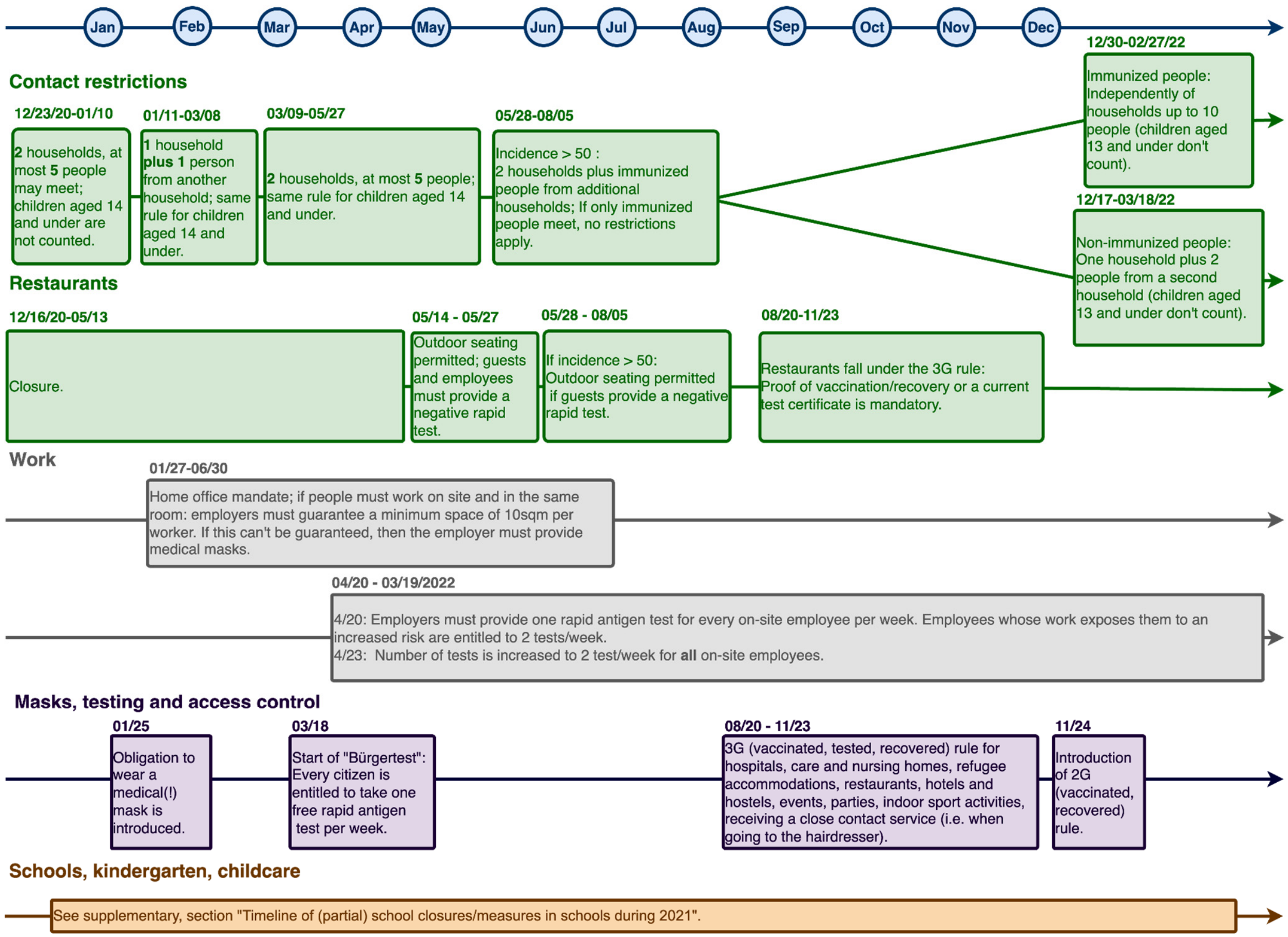

Figure S7: Timeline of introduced non-pharmaceutical interventions in Cologne in 2021. Sources for the collected data were the Coronaschutz-verordnungen and the Coronabetreuungsverordnungen by NRW as well as the national Arbeitsschutzverordnung. Contact restrictions and restrictions which target multiple contexts are colored in orange, while context-specific measures are colored in red. No claim to completeness is raised.

Attachment 1 to: *Hurraß J, Paltra S, Wolff A, Grüne B, Müller S, Kossow A, Nießen J, Nagel K. Identification of key COVID-19 infection contexts over time and the importance of within household infections. GMS Hyg Infect Control. 2026;21:Doc69. DOI: 10.3205/dgkh000678, URN: urn:nbn:de:0183-dgkh0006788*

References

1. MAGS NRW. Verordnung zum Schutz vor Neuinfizierungen mit dem Coronavirus SARS-CoV-2 im Bereich der Betreuungsinfrastruktur (Coronabetreuungsverordnung – CoronaBetrVO) - Vom 7. Januar 2021 [Internet]. 2021. Available from: https://www.land.nrw/media/dokument/corona-betreuungsverordnung-ab-11-januar

2. Statistisches Bundesamt. Kreisfreie Städte und Landkreise nach Fläche, Bevölkerung und Bevölkerungsdichte am 31.12.2020 [Internet]. 2021. Available from: https://www.destatis.de/DE/Themen/Laender-Regionen/Regionales/Gemeindeverzeichnis/Administrativ/04-kreise.html

3. RKI. COVID-19-Impfungen in Deutschland [Internet]. 2023. Available from: https://zenodo.org/record/7648451

4. Gesundheitsdirektion Kanton Zürich. Daten aus dem SARS-CoV-2 Contact Tracing im Kanton Zürich [Internet]. 2022 [cited 2022 May 12]. Available from: https://github.com/openZH/covid_19_contact_tracing_ZH

5. Buda S, an der Heiden M, Altmann D, Diercke M, Hamouda O, Rexroth U. Infektionsumfeld von erfassten COVID-19-Ausbrüchen in Deutschland. Epidemiologisches Bulletin [Internet]. 2020; Available from: https://edoc.rki.de/handle/176904/6944

6. RKI. Täglicher Lagebericht des RKI zur Coronavirus-Krankheit-2019 (COVID-19) - 13.07.2021 - Aktualisierter Stand für Deutschland [Internet]. 2021 [cited 2022 May 12]. Available from: https://www.rki.de/DE/Content/InfAZ/N/Neuartiges_Coronavirus/Situationsberichte/Jul_2021/2021-07-13-de.pdf?__blob=publicationFile

7. RKI. Robert Koch Institut. 2022 [cited 2022 Aug 10]. COVID-19-Fälle, die einem Ausbruch zugeordnet werden, nach Meldewoche und Infektionsumfeld. Available from: https://www.rki.de/DE/Content/InfAZ/N/Neuartiges_Coronavirus/Daten/Ausbruchsdaten.html

8. UK Health Security Agency. Weekly national Influenza and COVID-19 surveillance report - Week 19 report (up to week 18 data) - 12 May 2022 [Internet]. UKHSA; 2022 May. Available from: https://khub.net/documents/135939561/338928724/National+flu+and+COVID-19+surveillance+report+12+May+2022+week+19.pdf/6dacc73c-7887-dfcb-2e3a-a34308033d98

9. SAGE. SARS-COV-2: Transmission Routes and Environments, 22 October 2020 [Internet]. 2020 Oct [cited 2022 May 27]. Available from: https://www.gov.uk/government/publications/sars-cov-2-transmission-routes-and-environments-22-october-2020

10. The Luxembourg Government. COVID-19 cases - Weekly review: 2 - 8 May 2022 [Internet]. 2022 [cited 2022 May 12]. Available from: https://covid19.public.lu/en/news-covid19/communiques/2022/05/11-weekly-review.html

11. Lim C, Nam Y, Oh WS, Ham S, Kim E, Kim M, et al. Characteristics of transmission routes of COVID-19 cluster infections in Gangwon Province, Korea. Epidemiol Infect [Internet]. 2022 Jan 7;150:e19. DOI: 10.1017/S0950268821002788

12. Furuse Y, Sando E, Tsuchiya N, Miyahara R, Yasuda I, Ko YK, et al. Clusters of Coronavirus disease in communities, Japan, January-April 2020. Emerg Infect Dis [Internet]. 2020 Sep;26(9):2176–9. DOI: 10.3201/eid2609.202272

13. The national COVID-19 outbreak monitoring group. COVID-19 outbreaks in a transmission control scenario: challenges posed by social and leisure activities, and for workers in vulnerable conditions, Spain, early summer 2020. Eurosurveillance [Internet]. 2020 Sep 3 [cited 2022 May 12];25(35):2001545. DOI: 10.2807/1560-7917.ES.2020.25.35.2001545

14. Qian H, Miao T, Liu L, Zheng X, Luo D, Li Y. Indoor transmission of SARS-CoV-2. Indoor Air [Internet]. 2021 May;31(3):639–45. DOI: 10.1111/ina.12766

15. BMAS. SARS-CoV-2-Arbeitsschutzverordnung (Corona-ArbSchV) - Vom 21. Januar 2021 [Internet]. 2021. Available from: https://www.bundesanzeiger.de/pub/publication/5QH1uegEXs2GTWXKeln/content/5QH1uegEXs2GTWXKeln/BAnz%20AT%2022.01.2021%20V1.pdf?inline
